# Association of genetic and immuno-characteristics with clinical outcomes in patients with *RET*-rearranged non-small cell lung cancer: a retrospective multicenter study

**DOI:** 10.1186/s13045-020-00866-6

**Published:** 2020-04-15

**Authors:** Chang Lu, Xiao-Rong Dong, Jun Zhao, Xu-Chao Zhang, Hua-Jun Chen, Qing Zhou, Hai-Yan Tu, Xing-Hao Ai, Xiao-Feng Chen, Gai-Li An, Jun Bai, Jin-Lu Shan, Yi-Na Wang, Shuan-Ying Yang, Xiang Liu, Wu Zhuang, Hui-Ta Wu, Bo Zhu, Xue-Feng Xia, Rong-Rong Chen, De-Jian Gu, Hua-Min Xu, Yi-Long Wu, Jin-Ji Yang

**Affiliations:** 1 The Second School of Clinical Medicine, Southern Medical University, Guangzhou, China; 2 Guangdong Lung Cancer Institute, Guangdong Provincial People’s Hospital, Guangdong Academy of Medical Sciences, Guangzhou, China; 3 Cancer Center, Union Hospital, Tongji Medical College, Huazhong University of Science and Technology, Wuhan, China; 4 Key Laboratory of Carcinogenesis and Translational Research (Ministry of Education/Beijing), Department I of Thoracic Oncology, Peking University Cancer Hospital & Institute, Beijing, China; 5 Shanghai Lung Cancer Center, Shanghai Chest Hospital, Shanghai Jiao Tong University, Shanghai, China; 6 Oncology Department, The First Affiliated Hospital of Nanjing Medical University, Nanjing, China; 7 Department of Clinical Oncology, Shaanxi Provincial People’s Hospital, Xi’an, China; 8 Daping Hospital, Army medical center of PLA, Chongqing, China; 9 Department of Oncology, The First Affiliated Hospital of Zhejiang University, Hangzhou, China; 10 The Second Affiliated Hospital of Xi’an Jiaotong University, Xi’an, China; 11 Department of Cardiothoracic Surgery, The Second Affiliated Hospital, University of South China, Hengyang, China; 12 Fujian Provincial Cancer Hospital, Fuzhou, China; 13 Department of Oncology, Zhongshan Hospital, Xiamen University, Xiamen, China; 14 Institute of Cancer, Xinqiao Hospital, Third Military Medical University, Chongqing, China; 15 Geneplus-Beijing Institute, Beijing, China

**Keywords:** Advanced NSCLC, *RET* rearrangement, Next-generationsequencing, *TP53*, Immune checkpoint inhibitor

## Abstract

**Background:**

Rearranged during transfection (*RET*) has been proven to be a tumorigenic target in non-small cell lung cancers (NSCLCs). In *RET*-rearranged NSCLCs, molecular features and their impact on prognosis were not well illustrated, and the activity of mainstay therapeutics has not currently been well compared.

**Methods:**

Patients diagnosed with NSCLCs with *RET* rearrangements were analyzed for concomitant mutations, tumor mutation burden (TMB), PD-L1 expression, T cell receptor repertoire and clinical outcomes with chemotherapy, immune checkpoint inhibitors (ICIs), and multikinase inhibitors (MKIs).

**Results:**

Among 129 patients with *RET*-rearranged NSCLC who were analyzed, 41.1% (53/129) had co-occurring genetic alterations by next-generation sequencing, and concomitant *TP53* mutation appeared most frequently (20/53, 37.7%). Patients with concurrent *TP53* mutation (*n* = 15) had shorter overall survival than those without (*n* = 30; median, 18.4 months [95% CI, 8.6–39.1] vs 24.8 months [95% CI, 11.7–52.8]; *P* < 0.05). Patients with lower peripheral blood TCR diversity (*n* = 5) had superior overall survival compared with those with higher diversity (*n* = 6; median, 18.4 months [95% CI, 16.9–19.9] vs 4.8 months [95% CI, 4.5–5.3]; *P* = 0.035). An association with overall survival was not observed for PD-L1 expression nor for tumor mutation burden level. Median progression-free survival was not significantly different across chemotherapy, ICIs, and MKIs (median, 3.5 vs 2.5 vs 3.8 months). For patients treated with ICIs, the disease control rate was 60% (6/10) and the objective response rate was 20% (2/10).

**Conclusions:**

*RET*-rearranged lung cancers can be heterogeneous in terms of concomitant genetic alterations. Patients with concurrent *TP53* mutation or high peripheral blood TCR repertoire diversity have relatively inferior overall survival in this series. Outcomes with traditional systemic therapies in general are suboptimal.

## Background

The dawn of the targeted therapy era saw the discovery of receptor tyrosine kinase *RET* fusion in 1–2% of non-small cell lung cancers (NSCLC) [1, 2] and proved it to be tumorigenic and targetable. Regarding the tumorigenicity, although several studies reported the prevalence of concomitant genetic alterations based on a limited sample size [3–6], the effects of these concomitant alterations on clinical outcomes were scant.

Regarding the druggability, since more specific and potent TKIs targeting *RET* such as BLU-667 and LOXO-29 2[7–9] are currently not available for all of the patients, the common systemic treatment regimen now includes multikinase inhibitors (MKIs), chemotherapy, and immune checkpoint inhibitors (ICIs). The success of traditional MKIs is relatively limited [10–14]. The median progression-free survival (PFS) of the pemetrexed/platinum regimen was 19 months, 7.5 months, and 6.4 months in a single center [15], a Chinese cohort [5], and an international cohort [10], respectively. Although ICIs have been widely accepted, the outcomes of these treatment strategies in *RET*-altered patients have not currently been well compared, and the immuno-characteristics in those patients have also not been well characterized in previous studies [16, 17].

Here, we describe genetic and immune profiling in a multicenter cohort of patients with *RET*-rearranged NSCLC, analyze their associations with clinical outcomes, and document treatment outcomes in routine clinical care.

## Methods

### Study design and patients

The study flow chart is shown in Supplementary figure 1. This retrospective observational study was performed at 13 centers in China and included patients who had a pathologic diagnosis of NSCLC of any age with *RET* rearrangement determined by at least one of the validated tests including fluorescence in situ hybridization, reverse transcriptase polymerase chain reaction, and next-generation sequencing (NGS). Patients with acquired *RET* rearrangement after progression on *EGFR* TKIs were excluded due to the concern of the potential prognostic implications of frontline *EGFR*-TKI administration (*RET* cohort). This multicenter network of thoracic oncologists also identified *EGFR/ALK/ROS1*/pan-negative patients determined by targeted DNA sequencing performed in one institute (*EGFR/ALK/ROS1*/pan-negative cohort). There is no overlap between these cohorts. Only patients with locally advanced or metastatic NSCLC were analyzed for clinical outcomes. Written consent and institutional approval were obtained.

### Genotyping and immunotyping

Commercial targeted DNA sequencing (Geneplus or Burning Rock or Geneseeq) was used to calculate genetic alterations and tumor mutation burden (TMB). More than 90% of the samples were sequenced in one institute. Our genetic profiling platform is designed and validated to categorize point mutations, insertions, deletions, copy number variations, and rearrangements. PD-L1 levels by immunohistochemistry were assessed by one local laboratory as previously described [18].

#### DNA extraction and processing

Genomic DNA of tissue samples was extracted by using the QIAamp DNA FFPE Tissue Kit or the DNeasy Blood & Tissue Kit (Qiagen, Hilden, Germany). For cell-free DNA (cfDNA) extraction, plasma was separated by centrifugation at 1600×*g* for 10 min, then transferred to a new microcentrifuge tube and centrifuged at 16,000×*g* for another 10 min to remove any remaining cell debris. cfDNA was isolated from the plasma using the QIAamp Circulating Nucleic Acid Kit (Qiagen, Hilden, Germany). Peripheral blood lymphocytes (PBLs) were used to extract germline genomic DNA from each patient with the DNeasy Blood & Tissue Kit (Qiagen, Hilden, Germany). A Qubit fluorometer and the Qubit dsDNA HS (High Sensitivity) Assay Kit (Invitrogen, Carlsbad, CA USA) were used for DNA concentration measurement. And the size distribution of cfDNA was assessed with an Agilent 2100 BioAnalyzer and the DNA HS kit (Agilent Technologies, Santa Clara, CA, USA).

#### Library construction and target capture sequencing

We used protocols recommended in the Illumina TruSeq DNA Library Preparation Kit (Illumina, San Diego, CA) for the construction of the Indexed Illumina NGS libraries. About 20–80 ng cfDNA per sample was used. For genomic DNA extracted from either tissue or PBLs, about 1 μg DNA was sheared with a Covaris S2 ultrasonicator (Covaris, Woburn, MA, USA) to generate fragments with a peak of 250 bps for library construction. Then end repair, tailing, and ligation to the Illumina-indexed adapters were done according to the standard library construction protocol. The constructed libraries were hybridized to custom-designed biotinylated oligonucleotide probes (Roche NimbleGen, Madison, WI, USA) for target enrichment. The probes cover 1021 cancer-related genes (Supplementary table 1). The captured DNA fragments were amplified and pooled to generate multiplex libraries. Then sequencing was done using Illumina 2 × 75 bp paired-end reads with the HiSeq 3000 Sequencing System (Illumina, San Diego, CA).

#### Sequencing data analysis

After removing terminal adaptor sequences and low-quality reads, the clean reads were mapped and aligned to the reference human genome (hg19) with BWA (version 0.7.12-r1039) [19]. MuTect2 (3.4-46-gbc02625) [20] was used to call single nucleotide variants (SNVs) while GATK was employed to call small insertions and deletions (Indels). Copy number variations (CNVs) were detected using Contra (2.0.8) [21]. And structure variations (SVs) were detected with BreakDancer. All final candidate variants were verified with the integrative genomics viewer browser. TMB was defined as the number of somatic non-synonymous mutations per megabase including SNVs, insertions, and deletions of the panel region [22].

#### T cell receptor sequencing and data analysis

The T cell receptor (TCR) repertoire has recently emerged as a novel biomarker [23, 24]. Previous pilot studies showed that tumor-infiltrated TCR clonality [25] and peripheral blood T cell receptor repertoire diversity [26–28] could have a potential role as predictors of the response to ICI therapy. We conducted multiplex PCR amplification on complementarity-determining region 3 (CDR3), a hypervariable region of the TCR β chain that is unique to each TC R[29] as previously described [27]. The diversity metric accounts for both “richness” and “evenness” components, while richness is a measurement of the number of different specificities in the sample (e.g., the number of T cell clones with unique TCRs), evenness measures the relative abundance of these different specificities. Diversity can be measured in many ways; one of them uses Shannon’s entrop y[30], in which higher diversity values indicate a more diverse distribution of the receptor sequences. The evenness or relative abundance metric can be calculated in different manners, such as Pielou’s evenness, originally developed for measurements derived from ecology [31]. Clonality, a metric of T cell expansion and reactivity, ranges from 0 to 1 and describes the shape of the T cell frequency distribution: clonality values approaching 0 indicate a very even distribution of clone frequencies, whereas values approaching 1 indicate an increasingly asymmetric distribution in which a few clones are present at high frequencies [32]. The diversity and clonality of the tissue and blood TCR repertoire are represented T-Shannon, T-clonality, T-evenness, B-Shannon, B-clonality, and B-evenness, respectively, in this manuscript.

### Outcomes

Data on clinical treatments and outcomes were collected since advanced diagnosis. Overall survival was defined as the time between the date of advanced diagnosis to the date of death from any cause or last follow-up. PFS was measured from the start of treatment to disease progression, death from any cause or last follow-up. Disease control rate (DCR) was defined as the proportion of achieving disease control (stable disease or radiologically confirmed complete/partial response). The objective response rate (ORR) was defined as the proportion achieving an objective response (radiologically confirmed complete/partial response). The investigators and the treating physicians ascertained tumor response according to the Response Evaluation Criteria in Solid Tumors (RECIST) version 1.1 and iRECIST (a modified RECIST 1.1 for immune-based therapeutics). The median follow-up time was 12.7 months and the last follow-up date was June 28, 2019.

### Statistics

The data were analyzed by GraphPad Prism 8.0 and SPSS Statistics 19. A *P* value < 0.05 was considered statistically significant. The median TMB of *RET*-rearranged NSCLCs was compared with that of *ALK*-rearranged, *ROS1*-rearranged, *EGFR*-mutant NSCLCs from the screened population using the Mann-Whitney test. Patients, irrespective of ICI treatment in their disease course, were stratified by cut-off values of the Shannon, evenness, and clonality indexes. The cut-off values were determined by ROC analysis. Kaplan-Meier analysis was used to estimate PFS and overall survival, presented as median values. The log-rank test was used to compare the curves. Hazard ratios were calculated using the log-rank method. Spearman’s rank test was used to estimate correlations between the TCR repertoire indexes and overall survival. Baseline variables that were considered clinically relevant or that showed a univariate relationship with outcomes were entered into the multivariate Cox proportional hazards model (forward stepwise).

## Results

### Clinicopathologic and molecular characteristics

We retrospectively included 129 patients with *RET*-rearranged lung cancer from 13 centers. The majority of patients presented stage III–IV disease at initial diagnosis (*n* = 110, 85.3%). Patients were preponderantly never smokers (*n* = 58, 45.0%) and had adenocarcinoma histology (*n* = 112, 86.8%) with a median age of 57 years (range 24–82 years) and a sex makeup of 51.9% female and 48.1% male. The detailed clinical characteristics are provided in Table 1, and the frequencies of missing data are also shown.

**Table 1:**
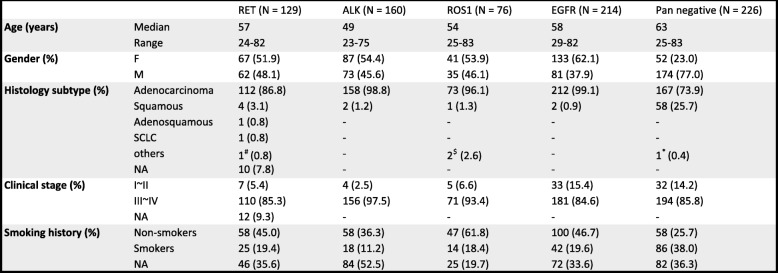
Clinicopathologic features in patients with *RET*-positive NSCLC. Demographics of 129 patients with *RET*-rearranged lung cancers as well as patients with *RET* wild-type lung cancers

**Fig. 1:**
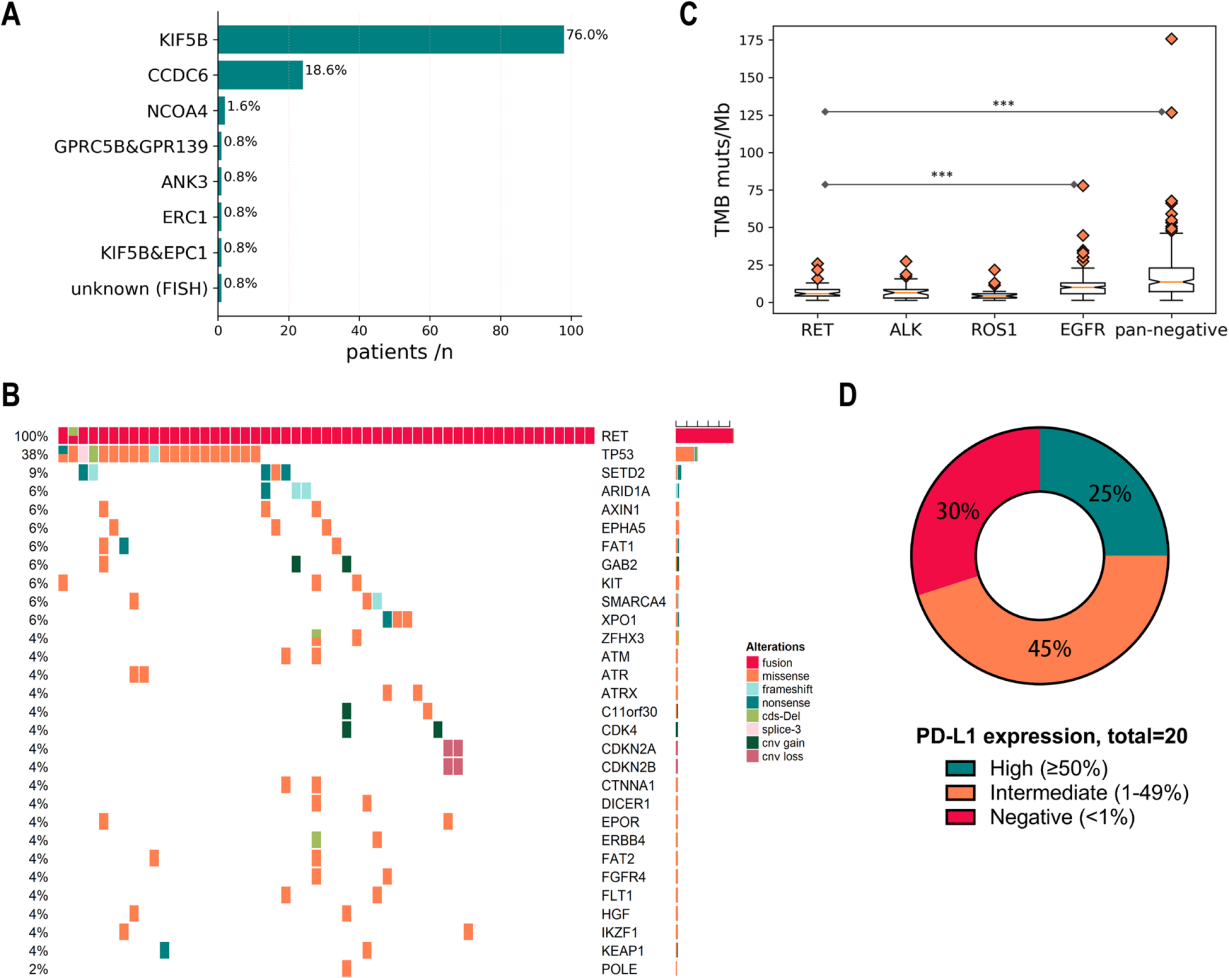
Genotype and immunophenotype of *RET*-rearranged lung cancers. **a***RET* upstream fusion partner. **b** Genomic alterations of patients with *RET*-positive NSCLC at the time they were diagnosed indicate that *RET*-rearrangement is mutually exclusive with common driver alterations. **c** Number of mutations of *RET*-rearranged lung cancers compared with *ALK/ROS1/EGFR*-altered lung cancers and *RET/ALK/ROS1/EGFR*-negative lung cancers. **d** PD-L1 expression in *RET*-positive NSCLC. Abbreviations: TMB - tumor mutation burden

Of 129 patients with baseline genetic profiles, all had *RET*-fusion by NGS or FISH, including 99 (76.0%) with *KIF5B-RET*, 24 (18.6%) with *CCDC6-RET*, and 2 (1.6%) with *NCOA4-RET*. No patient harbored concurrent driver oncogenic alterations. The most common concomitant mutations identified in *RET*-positive NSCLC were *TP53* (38%) and SETD2 (9%, Fig. 1a, b). Other co-alterations that have a potential impact on tumor biology include *KEAP1* (4%) and combined *CDKN2A/CDKN2B* (4%). TMB levels calculated based on a large panel of 1021 genes ranged from 1.4 to 25.9 muts/Mb (median = 5.8) in *RET*-rearranged NSCLCs. No significant difference in TMB level was found among alterations in *RET* and other fusion genes, such as *ALK* (range 1.4 to 27.4 muts/Mb, median = 6.5) and *ROS1* (ranged 1.4 to 21.6 muts/Mb, median = 4.3). TMB level appeared higher in *EGFR-mutant* NSCLCs (range 1.4 to 77.8 muts/Mb, median = 10.1) and pan-negative NSCLCs (range 1.4-175.7, media=13.7, p<0.0001 for both) (Fig. 1c). High (≥ 50%), intermediate (1–49%), and negative (< 1%) PD-L1 expression was observed in 5/20 (25%), 9/20 (45%), and 6/20 (30%) cases, respectively (Fig. 1d).

### Association of genetic and immuno-characteristics with clinical outcomes

**Fig. 2:**
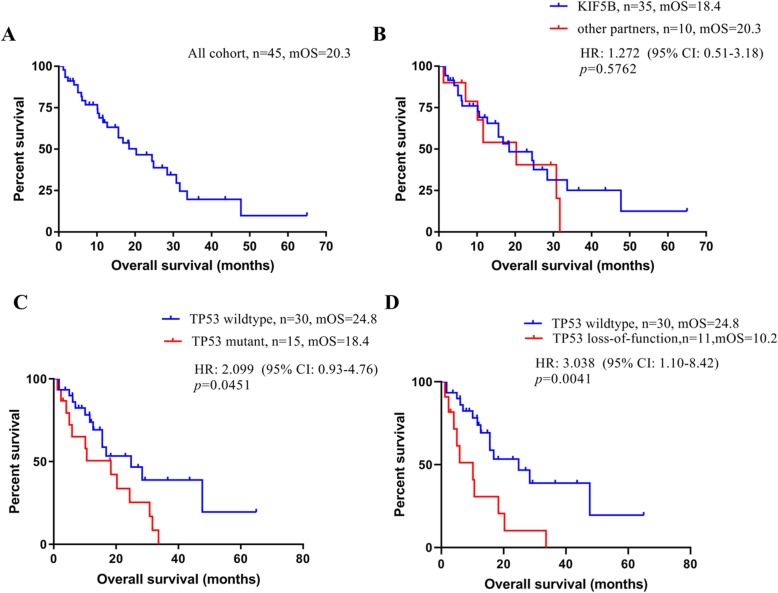
Survival of patients with advanced NSCLC. **a** Overall survival of all cohorts. **b** Overall survival by fusion partner. **c** Overall survival by *TP53* mutation status. Tick marks indicate censoring of the data at the last time the patient was known to be alive. **d** Overall survival of *TP53* loss-of-function and TP53 wildtype patients. Abbreviations: mOS - median overall survival

Analyses of overall survival were restricted to patients with advanced disease and those who had survival data (*n* = 45). In the entire cohort, the median overall survival from the initial advanced diagnosis was 20.3 months (95% CI, 8.4–49.2) (Fig. 2a). In cancers with known upstream fusion partners, *RET* fusions involving *KIF5B* (*n* = 35) were not associated with a benefit on overall survival compared to those involving partners other than *KIF5B* (*n* = 10; median overall survival, 18.4 months [95% CI, 7.8–43.5] vs 20.3 months [95% CI, 8.6–48.0]; *P* = 0.58) (Fig. 2b). We analyzed the overall survival of patients with ICI treatment in their disease course to assess the prognostic implications of TMB. The results were limited by the small number of patients (*n* = 6) (Supplementary figure 2). Patients with concurrent *TP53* mutation (*n* = 15) had shorter survival than those without (*n* = 30; median overall survival, 18.4 months [95% CI, 8.6–39.1] vs 24.8 months [95% CI, 11.7–52.8]; *P* < 0.05) (Fig. 2c). Notably, those harboring *TP53* loss-of-function (*n* = 11, including *TP53* loss-of-function and likely loss-of-function) showed a more obvious survival disadvantage (median overall survival, 10.2 months vs 24.8 months; *P* = 0.0041) (Fig. 2d, Supplementary table 2). Multivariable analysis of these patients revealed that concomitant *TP53* mutation was an independent poor prognostic factor (HR = 2.26 [95% CI, 1.04–4.91]; *P* = 0.040) (Table 2). An association with overall survival was not observed for PD-L1 expression.

**Table 2:**
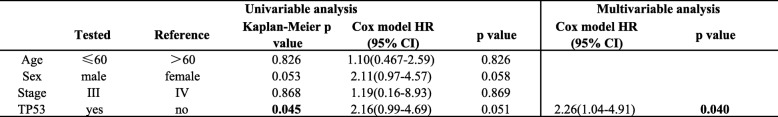
Multivariate analysis of overall survival

**Fig. 3:**
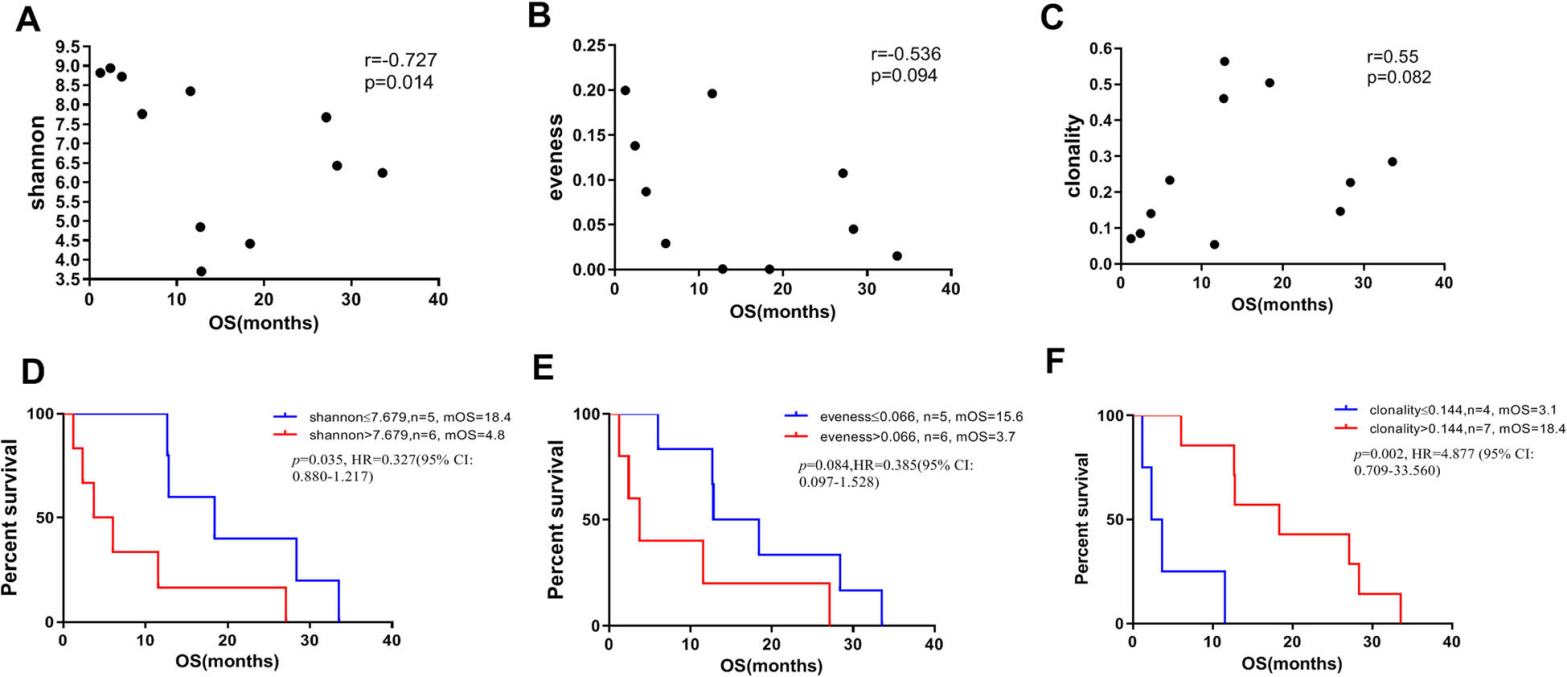
Peripheral blood T cell receptor repertoire associated with overall survival. Correlation of B-Shannon (**a**, **d**), B-evenness (**b**, **e**), and B-clonality (**c**, **f**) of TCR CDR3s with the overall survival from advanced diagnoses. Spearman’s rank test was used to estimate correlations between the TCR repertoire indexes and overall survival (**a**, **b**, **c**). Kaplan-Meier analysis was used to estimate overall survival, presented as median values. The cut-off values of TCR repertoire indexes were determined by ROC analysis. The log-rank test was used to compare the curves (**d**, **e**, **f**). Abbreviations: mOS - median overall survival

We collected peripheral blood (*n* = 19) and tissue samples (*n* = 22) from patients with advanced NSCLC to assess the diversity and clonality of the TCR repertoire. Of these patients, 21 with known overall survival from advanced diagnosis were analyzed to assess the potential prognostic significance of baseline blood (*n* = 11) or tissue TCR (*n* = 10) repertoire diversity. Patients, irrespective of ICI treatment in their disease course, were stratified by the cut-off value of each of three indexes determined by ROC analysis. A significant negative correlation was observed between overall survival and B-Shannon index (*P* = 0.014, Spearman *r* = − 0.727; Fig. 3a). Patients with a lower B-Shannon index or higher B-clonality were associated with significantly longer overall survival than those with a higher B-Shannon index or lower B-clonality (Fig. 3d, f). However, this association with overall survival was not found in other indexes (T-Shannon, T-clonality, T-evenness). In 24 samples with available data on treatment, baseline blood (*n* = 11) or tissue (*n* = 13) TCR repertoire diversity did not indicate a significant association with PFS.

### Outcomes with MKIs, ICIs, and chemotherapy

**Fig. 4:**
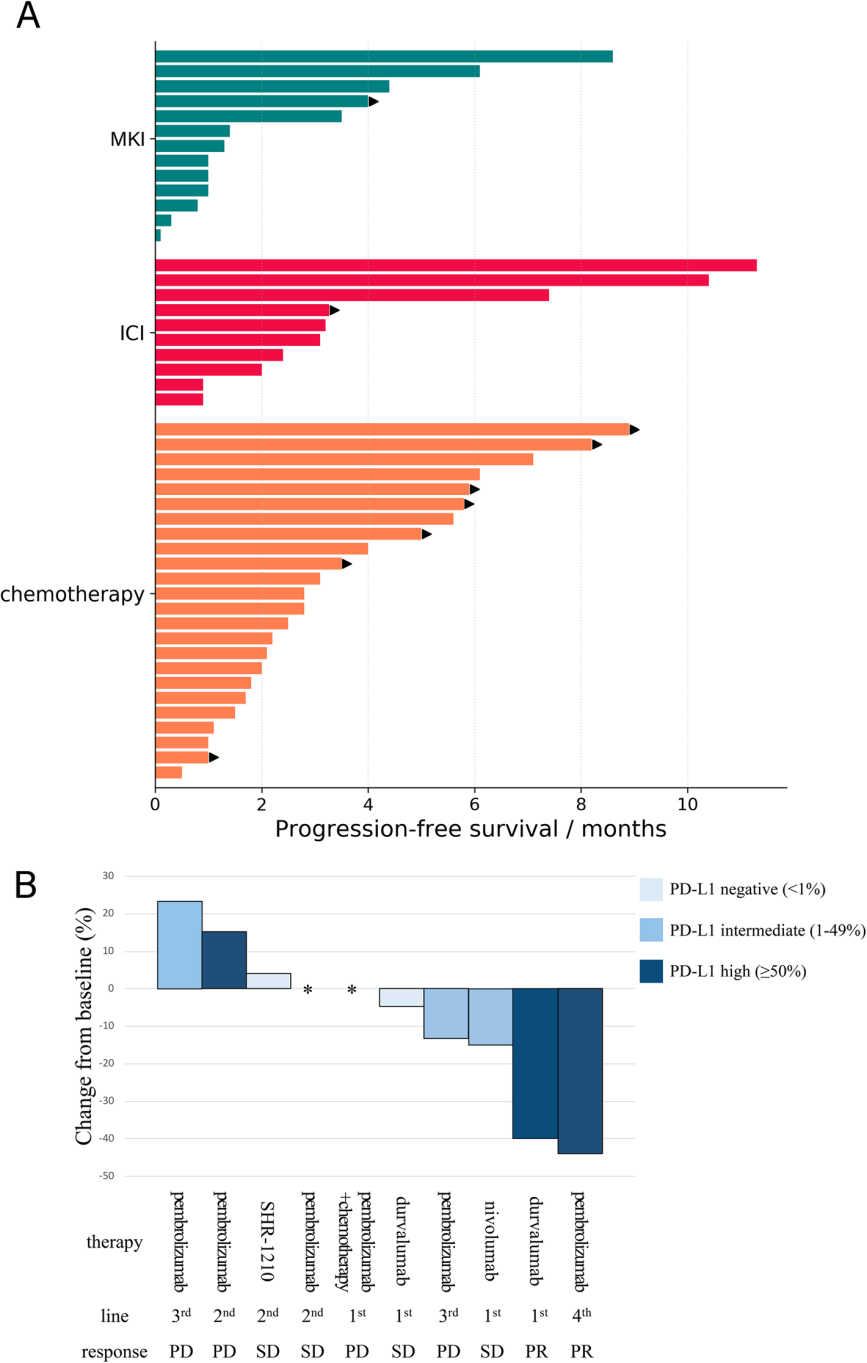
Outcomes with diverse therapies. **a** Swimmer plot of PFS across chemotherapy, ICI, and TKI (all of which are MKIs) cohorts. The MKI regimen included cabozantinib 80 mg qd, vandetanib 300 mg qd, anlotinib 12 mg qd, apatinib 500 mg qd, and vandetanib 300 mg+everolimus 5 mg qd. **b** Changes in target lesions from baseline to best response or the initial radiographic assessment, as well as treatment information (regimen, line, and response) and PD-L1 expression levels of 10 patients who received ICIs. Abbreviations: PFS - progression-free survival, ICI - immune checkpoint inhibitor, MKI - multi-kinase inhibitor, PD - progression disease, SD - stable disease, PR - partial response

After the advanced diagnosis, 45 patients received MKIs targeting *RET* (*n* = 19), ICIs (*n* = 11), and chemotherapy (*n* = 29). Twelve of 45 patients received two or three of these treatments in different lines during disease courses. Notably, none of our patients received selective *RET* inhibitors, owing to the unavailability of these drugs in our country by the date of data cut-off. PFS across the three treatment groups is shown in Fig. 4a and demonstrated no significant difference among the groups (median PFS, MKIs, 3.8 months [95% CI, 1.7–8.5], ICIs, 2.5 months [95% CI, 1.1–5.8], chemotherapy, 3.5 months [95% CI, 1.5–7.9]).

Eleven patients received ICIs in clinical trials or at their own expenses as first-line (*n* = 4), second-line (*n* = 4), and after second-line (*n* = 3) treatment. In 10 patients with evaluable response, DCR was 60%, and ORR was 20%. Three patients had durable PFS of 6.3, 10.4, and 11.5 months. Two of them were PD-L1 positive, while one patient lacked sample for IHC assessment. Changes in target lesions from baseline to best response, or the initial radiographic assessment, as well as treatment information of each patient (regimen, line, and response), are shown in Fig. 4b. The median tumor shrinkage/growth was − 2.4% (range − 44%~23.3%).

### Two cases with KIF5B-RET fusion and high level of PD-L1 expression responding to ICIs

**Fig. 5:**
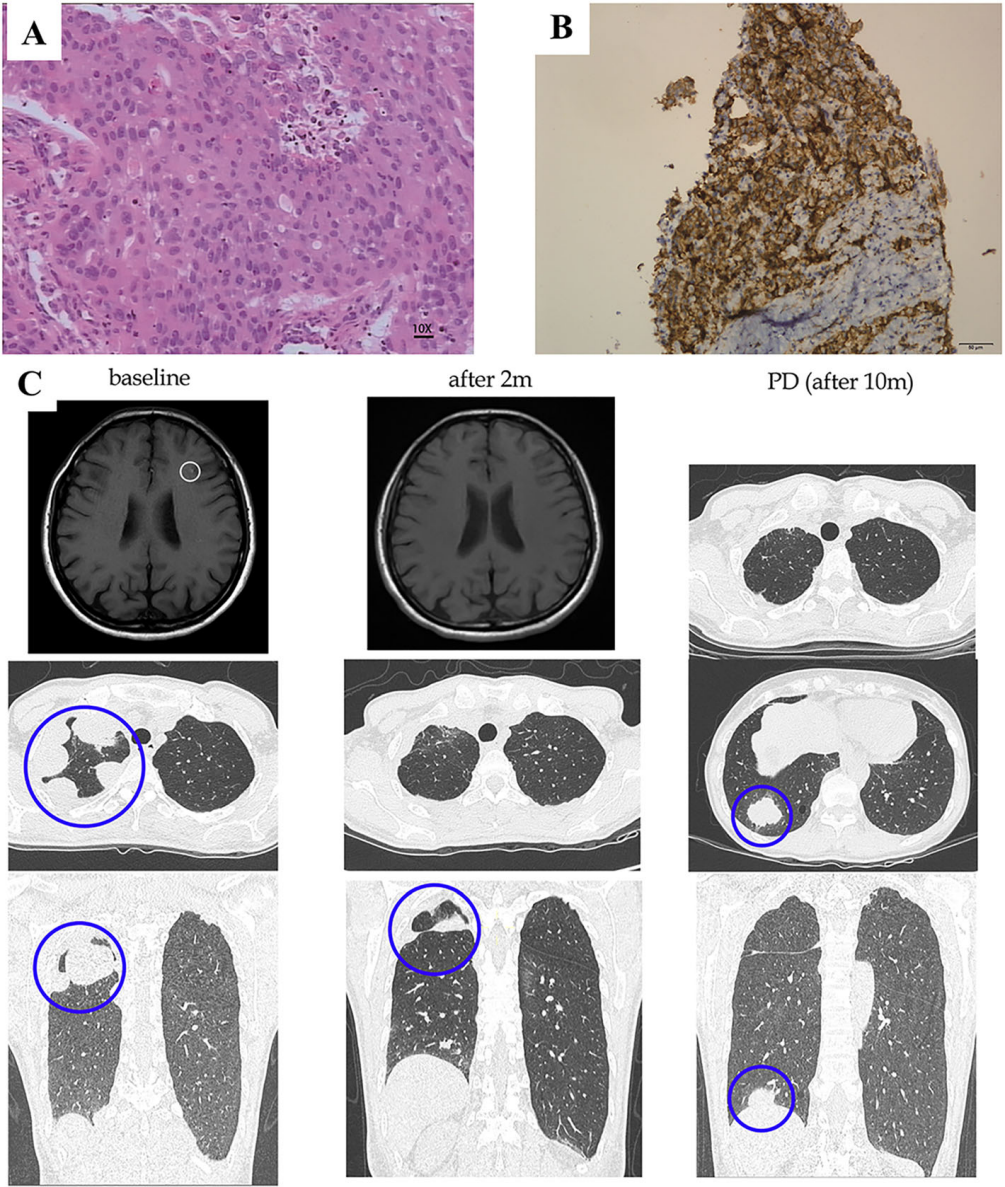
Response to pembrolizumab in a case with KIF5B-RET fusion and a high level of PD-L1 expression. **a** Adenocarcinoma nature (10×). **b** High level of PD-L1 expression (TPS = 50%). **c** Computed tomography scan and magnetic resonance imaging revealing the clinical response to pembrolizumab. Abbreviations: PD - progression disease

Case 1 was a 55-year-old female never smoker, with *KIF5B-RET*-rearranged lung adenocarcinoma and brain metastasis, was treated with second-line cabozantinib. Four months later, intrathoracic progression was observed. Rebiopsy revealed lung adenocarcinoma with *KIF5B-RET* fusion and a high level of PD-L1 expression (TPS = 50%; Fig. 5a, b). She was advised to start pembrolizumab thereafter. The patient achieved a confirmed partial response evaluated according to iRECIST and tumor reduction, both intrathoracic and intracranial PR, was noted (Fig. 5c).

Case 2 was a 74-year-old male with *KIF5B-RET*-rearranged stage IVA (cT1bN3M1b) lung adenocarcinoma. Baseline tissue showed a high level of PD-L1 expression (TPS > 50%). He was treated with first-line durvalumab and had confirmed partial response as the best response. Treatment is ongoing at the date of data cut-off.

## Discussion

Despite the rarity of this driver gene, we report a relatively large sample of multicenter patients with RET-altered NSCLC with therapies commonly used in clinical practice. We demonstrate that this group of lung cancers is characterized by heterogeneous genotype and PD-L1 expression, as well as low TMB. Patients harboring concomitant *TP53* were associated with inferior overall survival. We also found prognostic significance of TCR repertoire diversity in the peripheral blood. Although a subgroup of patients could benefit from ICIs, the optimal treatment option in routine clinical care remains to be defined.

In this study, we found an unfavorable clinical outcome in *RET/TP53* co-mutated patients. Compared with other genes, *TP53* co-mutations occur rather frequently with *RET* fusions. Recent works have suggested a negative impact of *TP53* mutations on the outcomes of patients with *EGFR*-mutant [33, 34] and *ALK*-rearranged NSCLC [35, 36]. However, in *RET*-positive lung cancers, concomitant *TP53* mutations have not been described as poor prognostic factors. The negative prognostic effect of *TP53* mutations might be attributed to their tumor-suppressive function loss, genomic instability function gain, and abilities of cancer cell transcriptome and phenotype regulation [37]. Future research is warranted to improve the outcomes. In addition to *TP53*, other co-mutated genes, such as *PIK3CA*, etc., are also detected. All these findings call for an intensive study of the role of these additional genetic abnormalities in disease evolution and how they might influence the efficacy of treatments.

Whether *RET* fusion is mutually exclusive with other oncogenic drivers remains controversial. Recently, a study analyzing the fusion landscape in 33 cancer types highlighted the generally mutual exclusivity between fusions and mutations [38]. Nonetheless, Wang et al. [39] reported that one unique mutational signature in Chinese patients with NSCLC is associated with an increasing *EGFR* mutation rate together with gene fusions, such as *RET* and *ALK*. In one retrospective analysis, concurrent *EGFR* mutations were found in 7 of 47 *RET*-rearranged adenocarcinomas [3]. In our study, patients with acquired RET-rearrangement after progression on EGFR TKIs were excluded due to the concern of the potential prognostic implications of frontline EGFR-TKI administration, and no co-existence of other driver-gene alteration appeared.

Previous studies have shown TCR repertoire diversity in the peripheral blood to be an indicator of prognosis, and high TCR repertoire diversity might indicate favorable outcomes [23, 27, 28]. Our study supplemented the prognostic value of TCR repertoire diversity in *RET*-driven lung cancers but disagree with the latter notion. Two explanations should be considered. First, although tumors with high TCR repertoire diversity are interpreted as biologically hot, two recent studies have indicated that intrinsic tumor reactivity of the intratumoral TCR repertoire of CD8 T cells can be limited and variable, and there are bystander CD8 T cells [40, 41]. Therefore, it seems that not all T cells are specific for tumor antigens in this study. Second, our data are limited in the dynamic analysis of the TCR repertoire during treatment and tumor evolution. As T cells can be easily isolated from patients’ blood without losing much of their functions [24], TCR repertoire analysis can be utilized to stratify patients with long survival or screen ICI candidates in the future.

Importantly, our DCR of ICIs is superior to that presented before (60% vs. 25%), while PD-L1 expression is similar [17, 42]. Although patients with selected druggable tumor alterations were considered as poor candidates for ICIs (for example, *EGFR*-mutant and *ALK*-rearranged lung cancers), and diverse efficacy of ICIs in *RET*-positive patients was reported in previous studies [16], a subgroup of patients exists who can benefit from ICIs as shown in our study. The challenge is how to precisely select these patients in future exploration. In this study, two patients with high PD-L1 expression experienced a satisfying response to ICIs. In a previous study, *CCDC6-RET* was found to be immunogenic because of its peptide level [38]. Predictive immuno-biomarkers are critical. Studies on overall immunogenicity and immune landscape are indispensable to strengthen the full understanding of ICIs in cancers with driver gene alterations.

However, the DCR of ICIs in our study seems to be driven by patients whose best objective response to this treatment was stable disease (4/6), and the median PFS is relatively short, revealing suboptimal outcomes of immune checkpoint inhibition. Notably, only one case received pemetrexed plus pembrolizumab as first-line therapy, a combination approved by the FDA based on data from the phase III KEYNOTE-189 trial, but yielded no response. Thus, considering evidence from several studies focusing on ICIs in oncogene-addicted NSCLCs, recently a summary of a multidisciplinary roundtable discussion recommended that ICIs should currently only be considered after exhaustion of targeted therapies and chemotherapies in these patients [43].

Our observations can generate meaningful implications for clinical trial settings. Currently, all clinical trials of first-line ICIs, either single- or dual-agent, have excluded *EGFR*-mutant and *ALK*-rearranged lung cancers but included patients with *RET*-rearranged lung cancers. In our study, a trend towards inferior outcomes was observed in ICIs compared with chemotherapy. Thus, patients with *RET* rearrangement might not always be appropriate for first-line immunotherapy trials; they should consider the use of selective targeted therapies (if possible, since more specific and potent TKIs targeting *RET* are unavailable for most of our patients at the moment, and the efficacy of MKIs is disappointing) and chemotherapy instead until more specific biomarkers are found to distinguish responders and nonresponders to immunotherapy.

Our study has several limitations. First, dynamic changes in the TCR repertoire are lacking. Next, potential intratumor heterogeneity, evolution during the disease course, and treatment were not addressed by multiregional NGS. Future analysis of NGS data from larger databases is warranted. Moreover, our findings were limited to the relatively small sample size of patients with available treatment data and overlapping in each treatment group.

## Conclusions

In summary, in addition to confirmation of *RET*-positive lung cancer heterogeneous genotypes and immunotypes, we first reported that patients with concurrent *TP53* mutations or high TCR repertoire diversity have relatively unfavorable outcomes. Outcomes with traditional systemic therapies in general are suboptimal. More work is required to understand the biology of *RET*-rearranged lung cancers and to tailor therapeutic strategies.

## Supplementary information

**Additional file 1. **Supplementary table 1. **The 1021 panel.**

**Additional file 2. **Supplementary figure 1. **Study flow chart.**

**Additional file 3. **Supplementary table 2. **TP53 mutation details.**

**Additional file 4. **Supplementary figure 2. **Overall survival of patients by tumor mutation burden (TMB) status.**

## Data Availability

The datasets used and analyzed during the current study are available from the corresponding authors on reasonable request.

## Abbreviations

CDR: Complementarity-determining regions
DCR: Disease control rate
ICI: Immune checkpoint inhibitor
MKI: Multikinase inhibitors
NGS: Next-generation sequencing
NSCLC: Non-small cell lung cancers
ORR: Objective response rate
PBL: Peripheral blood lymphocyte
PFS: Progression-free survival
RECIST: Response Evaluation Criteria in Solid Tumors
RET: Rearranged during transfection
TCR: T cell receptor
TMB: Tumor mutation burden
