# Supplementary figures and images for: Association of genetic and immuno-characteristics with clinical outcomes in patients with RET-rearranged non-small cell lung cancer: a retrospective multicenter study
Source: J Hematol Oncol. 2020 Apr 15;13:37. doi: 10.1186/s13045-020-00866-6 (PMC7160902; doi:10.1186/s13045-020-00866-6)

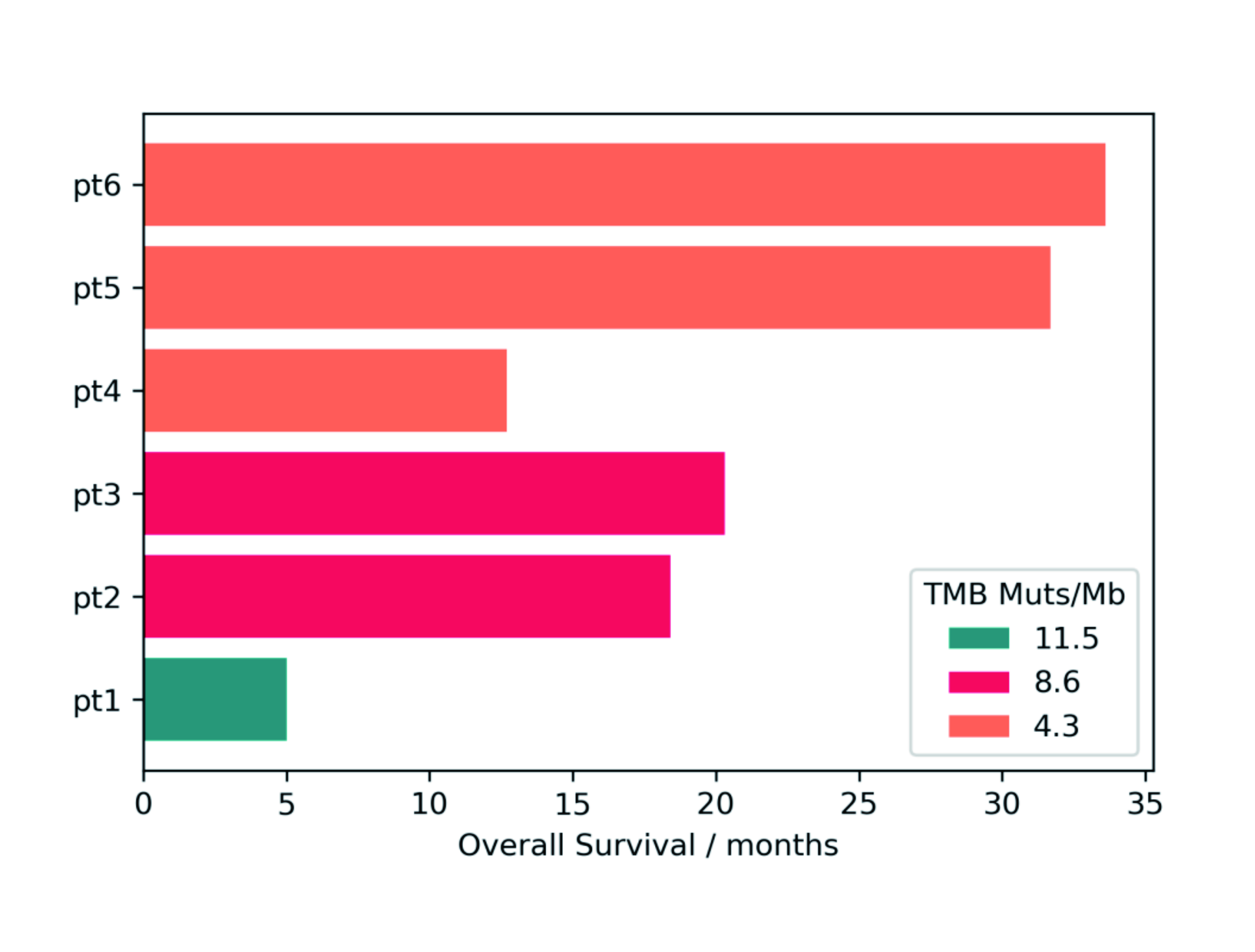

Supplement: Supplementary file 4 — Additional file 4. Supplementary figure 2. Overall survival of patients by tumor mutation burden (TMB) status. [file 13045_2020_866_MOESM4_ESM.tif]
